# Local Applications of Myostatin-siRNA with Atelocollagen Increase Skeletal Muscle Mass and Recovery of Muscle Function

**DOI:** 10.1371/journal.pone.0064719

**Published:** 2013-05-22

**Authors:** Emi Kawakami, Nobuhiko Kawai, Nao Kinouchi, Hiroyo Mori, Yutaka Ohsawa, Naozumi Ishimaru, Yoshihide Sunada, Sumihare Noji, Eiji Tanaka

**Affiliations:** 1 Department of Orthodontics and Dentofacial Orthopedics, Institute of Health Biosciences, The University of Tokushima Graduate School, Tokushima, Japan; 2 Department of Neurology, Kawasaki Medical School, Okayama, Japan; 3 Department of Oral Molecular Pathology, Institute of Health Biosciences, The University of Tokushima Graduate School, Tokushima, Japan; 4 Department of Life Systems, Institute of Technology and Science, The University of Tokushima, Tokushima, Japan; University of Pittsburgh, United States of America

## Abstract

**Background:**

Growing evidence suggests that small-interfering RNA (siRNA) can promote gene silencing in mammalian cells without induction of interferon synthesis or nonspecific gene suppression. Recently, a number of highly specific siRNAs targeted against disease-causing or disease-promoting genes have been developed. In this study, we evaluate the effectiveness of atelocollagen (ATCOL)-mediated application of siRNA targeting *myostatin* (*Mst*), a negative regulator of skeletal muscle growth, into skeletal muscles of muscular dystrophy model mice.

**Methods and Findings:**

We injected a nanoparticle complex containing myostatin-siRNA and ATCOL (Mst-siRNA/ATCOL) into the masseter muscles of mutant caveolin-3 transgenic (mCAV-3Tg) mice, an animal model for muscular dystrophy. Scrambled (scr) -siRNA/ATCOL complex was injected into the contralateral muscles as a control. Two weeks after injection, the masseter muscles were dissected for histometric analyses. To investigate changes in masseter muscle activity by local administration of Mst-siRNA/ATCOL complex, mouse masseter electromyography (EMG) was measured throughout the experimental period via telemetry. After local application of the Mst-siRNA/ATCOL complex, masseter muscles were enlarged, while no significant change was observed on the contralateral side. Histological analysis showed that myofibrils of masseter muscles treated with the Mst-siRNA/ATCOL complex were significantly larger than those of the control side. Real-time PCR analysis revealed a significant downregulation of *Mst* expression in the treated masseters of mCAV-3Tg mice. In addition, expression of myogenic transcription factors was upregulated in the Mst-siRNA-treated masseter muscle, while expression of adipogenic transcription factors was significantly downregulated. EMG results indicate that masseter muscle activity in mCAV-3Tg mice was increased by local administration of the Mst-siRNA/ATCOL complex.

**Conclusion:**

These data suggest local administration of Mst-siRNA/ATCOL complex could lead to skeletal muscle hypertrophy and recovery of motor disability in mCAV-3Tg mice. Therefore, ATCOL-mediated application of siRNA is a potential tool for therapeutic use in muscular atrophy diseases.

## Introduction

Small interfering RNA (siRNA) can degrade complementary mRNA by RNA interference (RNAi), a process of sequence-specific, posttranscriptional gene silencing active in plants and animals [1], [2]. Because the gene silencing effect of siRNA is potent and sequence-specific, siRNA has been applied as a powerful tool to suppress targeted gene expression and as a promising therapeutic molecule against diseases, including cancer [3], [4].

Due to safety issues, non-viral systems of siRNA delivery are preferred [5], [6]. Atelocollagen (ATCOL), a pepsin-treated type I collagen lacking the telopeptides at the N and C terminals that confer antigenicity, has been shown to efficiently deliver chemically unmodified siRNAs to metastatic tumors in vivo [7], [8]. Based on its practical use as an siRNA delivery platform, we previously adapted an ATCOL-mediated oligonucleotide system to deliver a myostatin-targeting siRNA into skeletal muscle and found that local and systemic administration of myostatin-targeting siRNA coupled with ATCOL led to a marked stimulation of muscle growth in vivo within a few weeks [9], [10]. However, possible alterations of muscle function have not been investigated in these studies.

Myostatin, also called growth differentiation factor 8 (GDF8), is a member of the transforming growth factor-β (TGF-β) superfamily and its expression is almost exclusively restricted to the skeletal muscle lineage [11]. Transgenic mice expressing myostatin containing a missense mutation showed a large and widespread increase in skeletal muscle mass as a result of muscle hyperplasia without hypertrophy [12]. Meanwhile, myostatin null mice exhibited a two-fold increase in skeletal muscle mass compared to controls, which results from both muscle hyperplasia and hypertrophy [11], [13]. The difference in muscle mass between the dominant negative myostatin and null myostatin mice likely results from inhibitory levels of endogenous myostatin proteins. Therefore, myostatin downregulation may serve as a potentially important mechanism for treating diseases associated with muscle wasting, such as muscular dystrophy.

We previously demonstrated that myostatin inhibition induced by overexpression of the myostatin pro-domain prevented muscular atrophy and normalized intracellular myostatin signaling in a mouse model of limb-girdle muscular dystrophy 1C (LGMD1C) [14]. Furthermore, myostatin inhibition also suppressed muscular atrophy in caveolin-3-deficient mice that expressed a dominant-negative form of the caveolin-3 gene [14]. The aim of this study is to evaluate the effectiveness of ATCOL-mediated local administration of myostatin-targeting siRNA into skeletal muscles in LGMD1C model mice, and to evaluate this method as a future treatment for muscular dystrophy. We hypothesize that skeletal muscle behavior will be changed due to the alteration of muscle mass and function induced by the local administration of myostatin-targeting siRNA/ATCOL (Mst-siRNA/ATCOL) into the skeletal muscles of LGMD1C model mice.

## Results

### Effects of local administration of Mst-siRNA/ATCOL complex in masseter muscles

**Figure 1:**
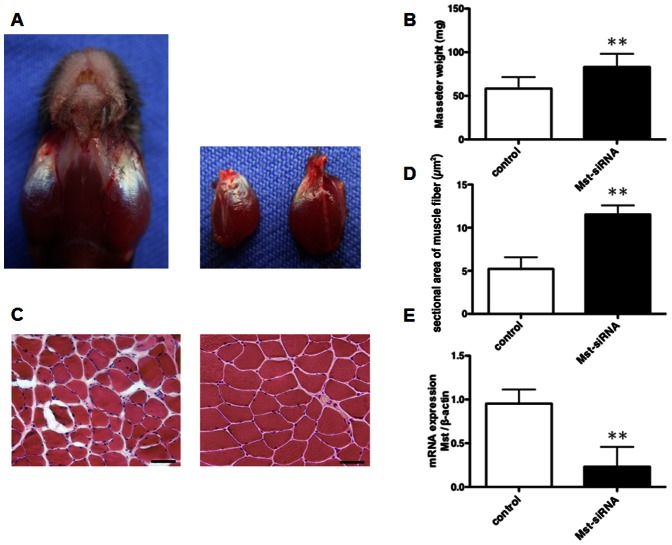
Local administration of Mst-siRNA/ATCOL complex causes an enlargement of the masseter muscle in the mCAV-3Tg mouse. (A) Photographs of siRNA-treated muscles. The left muscle injected with the Mst-siRNA/ATCOL complex show a marked increase in muscle mass compared to the right muscle injected with the control siRNA. (B) Average muscle weights. The muscle weight of the Mst-siRNA-treated masseter muscle is significantly larger than that of the control muscle. (C) Hematoxylin and eosin staining of the control and Mst-siRNA-treated masseter muscles. Scale bars = 50 µm. (D) Average cross-sectional areas. The sectional area of fiber is significantly larger in the Mst-siRNA-treated masseter muscle than in the control. (E) The ratio of the amount of myostatin mRNA for the masseter muscles. The mRNA expression level in the Mst-siRNA-treated masseter muscle is significantly higher than that in the control masseter muscle. Graphed data are expressed as mean ± SD. ** p<0.01, n = 12.

Two weeks after injection of Mst-siRNA/ATCOL complex, we dissected the muscle tissue and observed the gross morphology of the masseter muscles. The Mst-siRNA/ATCOL-treated masseter muscle was enlarged, while no significant change was observed on the contralateral control side (Fig. 1A). The Mst-siRNA/ATCOL-treated masseter muscles weighed significantly more than those treated with scrambled (scr) -siRNA/ATCOL (p<0.01, n = 12, Fig. 1B).

Histological analysis showed that the myofibril sizes of the Mst-siRNA/ATCOL-treated masseter muscles were larger than those of the contralateral muscles treated with control siRNA/ATCOL (Fig. 1C). Examining the fiber sizes of 200 myofibers per group, the population of fiber sectional area indicated a shift from smaller to larger fibers in the Mst-siRNA/ATCOL-treated muscles. The average cross-sectional area of muscle fiber treated with Mst-siRNA/ATCOL was about 2.5 times larger than that of control (p<0.01, n = 12), indicating muscle hypertrophy (Figure 1D). To confirm that local administration of Mst-siRNA/ATCOL reduced *myostatin* mRNA levels in the masseter muscles, we determined the ratio of *myostatin* mRNA levels in the siRNA-treated muscle to *myostatin* mRNA present in contralateral muscle. The average treated/untreated ratio in the bilateral siRNA-treated muscles was 0.23±0.35 (p<0.01, n = 12), representing a significant reduction in *myostatin* mRNA (Fig. 1E).

**Figure 2:**
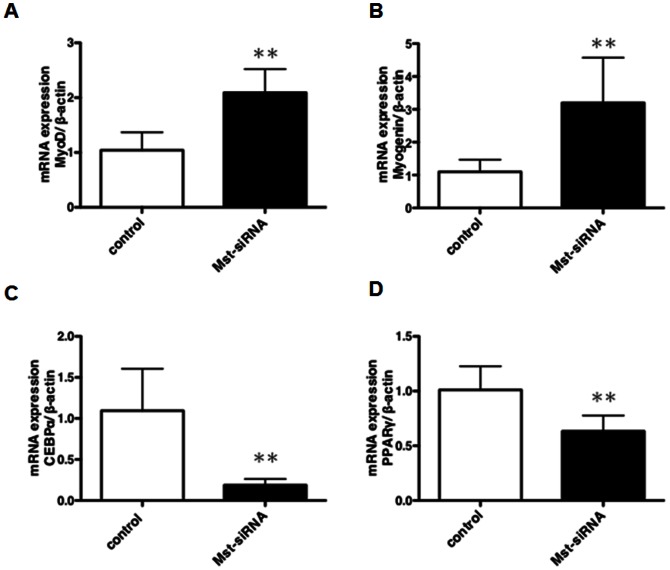
Effect of local administration of the Mst-siRNA/ATCOL complex on the gene expression levels of *MyoD*, *myogenin*, *PPARγ* and *CEBPα*. The expression levels were first calculated as a ratio to *GAPDH* expression levels. Subsequently, the ratios of these genes were averaged to give the mean values indicated in the graphs. The data are expressed as the mean ± SD. ** p<0.01, n = 12. (A) *MyoD*, (B) *Myogenin*, (C) *PPARγ*, (D) *CEBPα*.

To examine the effect of ATCOL-mediated local transfer of Mst-siRNA on myogenic differentiation, the mRNA levels of the transcription factors, *MyoD*, *myogenin*, *PPARγ* and *CEBPα* were estimated using real-time quantitative PCR (Fig. 2). Compared to controls, Mst-siRNA/ATCOL-treatment significantly (p<0.01) upregulated mRNA expression of the myogenic regulatory factors *MyoD* and *myogenin*, which in turn promoted skeletal muscle formation (Fig. 2A, B). Meanwhile, mRNA expression of *CEBPα* and *PPARγ*, adipogenesis transcription factors, were significantly downregulated by local administration of the Mst-siRNA/ATCOL complex, which may indicate a decrease in mesenchymal lineage cell differentiation to adipocyte (p<0.01, Fig. 2C, D).

### Electromyographic findings

**Figure 3:**
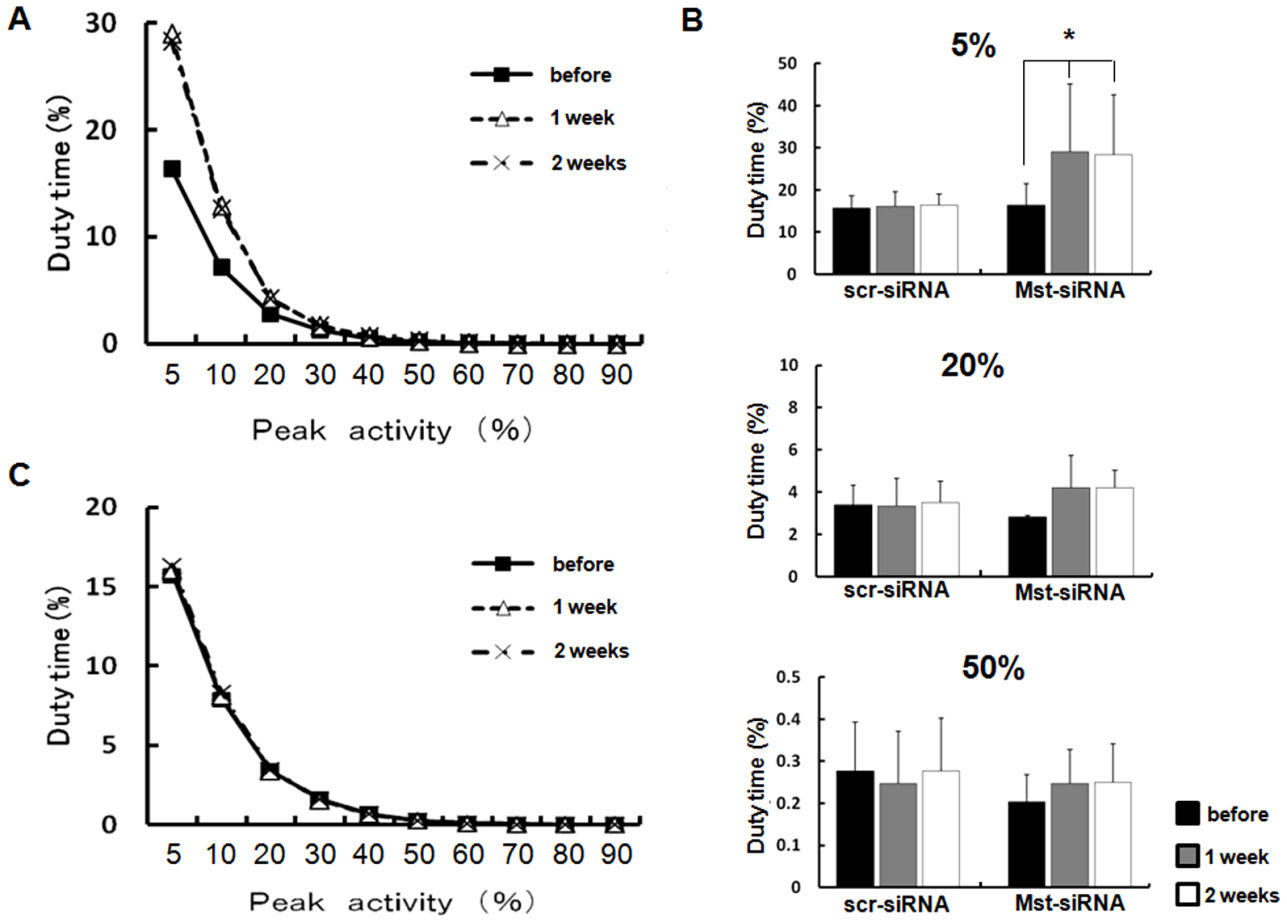
Daily duty times of the masseter muscles before and 1 and 2 weeks after Mst-siRNA/ATCOL complex administration. (A) The duty times, at various activity levels, of right masseter muscle in one animal before and 1 and 2 weeks after Mst-siRNA/ATCOL administration. (B) The duty times of the masseter muscle at exceeding 5, 20 and 50% of the peak activity level. At 1 and 2 weeks after local administration of Mst-siRNA, the duty times are significantly larger than before administration, while the duty times of the scr-siRNA-treated masseter muscle reveals no or less changes. * p<0.05, n = 4. (C) The duty times, at various activity levels, of left masseter muscle in one animal before and 1 and 2 weeks after scr-siRNA/ATCOL administration.

Representative daily duty times of the masseter muscle of one animal before Mst-siRNA/ATCOL complex administration, as well as 1 and 2 weeks after Mst-siRNA/ATCOL complex administration, are shown in figure 3A. In spite of the recording time, duty times were highest for activities exceeding 5% of the peak EMG and declined rapidly with increasing activity level. Before Mst-siRNA/ATCOL complex administration, the average duty time for activities exceeding 5% of the peak EMG level was 16.4±4.0% (Fig. 3B). At 1 week after the administration of Mst-siRNA/ATCOL, the duty time showed a significant increase (p<0.05, n = 4) to 29.0±16.2% compared to before Mst-siRNA/ATCOL administration (Fig. 3B). Furthermore, even at 2 weeks after Mst-siRNA/ATCOL complex administration, the Mst-siRNA/ATCOL-treated masseter muscle showed similar duty time for activity exceeding 5% of the peak EMG level, which was also significantly larger than the duty time before Mst-siRNA/ATCOL administration (p<0.05, Fig. 3B). Meanwhile, the duty times for activities exceeding 20% and 50% of the peak EMG level of the myostatin-siRNA-treated masseter showed a slight but insignificant increase compared to before Mst-siRNA/ATCOL administration. Duty time levels for activities exceeding both 20% and 50% were kept even at 2 weeks after Mst-siRNA/ATCOL administration. On the other, the duty times, at various activity levels, of the masseter muscle with scr-siRNA/ATCOL administration revealed almost no changes through the experimental period (Fig. 3B, C).

## Discussion

Duchenne muscular dystrophy (DMD), an X-linked, recessive disorder, is the most common and severe form of childhood muscular dystrophy [15], [16], which is caused by mutations in the *dystrophin* gene [17], [18]. It is a severe muscle wasting disorder that affects 1/3500 male births [19]. The mdx mouse, a well-known DMD model, has been used for previous studies of muscular wasting disease. Linkage studies, absence of dystrophin, and reduced levels of message indicate that the mutation in mdx mice lies in the gene for *dystrophin*, the gene that is defective in DMD [20]. However, these mice are characterized by early onset muscle degeneration and very mild clinical disease. In mdx mice, the disease is minimally progressive, and muscle fibrosis is absent [20], [21]. As a result, the mdx mouse likely exhibits insufficient muscular dystrophy in both histological and functional aspects to accurately model DMD [20], [21]. By contrast, the mCAV-3Tg mouse used in this study was developed as a limb girdle muscular dystrophy 1C (LGMD1C) model [22]. The skeletal muscle phenotype of this transgenic mouse showed severe myopathy, with loss of caveolin-3, resulting in muscle fiber degeneration, motor deficits and disability [14], [22]. As the aim of this study is to examine the effectiveness of ATCOL-mediated administration of myostatin-targeting siRNA into skeletal muscles as a future treatment remedy of muscular dystrophy, we chose the mCAV-3Tg mouse as a muscle dystrophy model.

Our results show that the local administration of the Mst-siRNA/ATCOL complex into the masseter muscle of the mCAV-3Tg mouse induced an increase in muscle mass and an enlargement of myofibril sizes, as shown previously in the masseter muscles of wild type and mdx mice [9]. Zhu et al. [23] generated a dominant negative myostatin by introducing a mutation at the cleavage site and reported that these mice exhibited a 20–35% increase in muscle mass that resulted from myofiber hypertrophy but not hyperplasia. Furthermore, mice fully null for myostatin revealed a double increase in skeletal muscle mass compared to normal muscle, resulting from both muscle hyperplasia and hypertrophy [11], [13]. Postnatal skeletal muscle growth is generally ascribed to enlargement of existing muscle fibers rather than to cellular proliferation [24]. Therefore, the increase in masseter muscle mass induced by myostatin-siRNA administration might result from myofiber hypertrophy. However, we also showed a significant upregulation of *MyoD* and *myogenin* mRNA expression after myostatin-siRNA application. MyoD, a protein with a key role in regulating muscle differentiation, is known as one of the earliest markers of myogenic commitment and is expressed in activated satellite cells [25]. Satellite cells in skeletal muscle proliferate and give rise to new myoblastic cells (i.e., immature muscle cells) [24]. Magee et al. [26] demonstrated that in skeletal muscle transfected with myostatin shRNA plasmid satellite cell number was increased by over 2-fold. Therefore, the increased masseter muscle mass we observed after myostatin-siRNA administration may not only be a result of hypertrophy but hyperplasia as well, although the increase in the number of fibers in the masseter muscle is relatively small.

In the present study, histological analysis shows that the masseter muscles of the CAV-3Tg mouse have increased intramuscular connective tissue, although this is not adipose tissue. A replacement of muscle mass by adipose cells is seen in human DMD, in which the loss of viable myofibers is accompanied by an expansion of adipose mass within the muscle [27]. Since intramuscular connective tissue is a potential contributor to declining force production [28], muscle weakness in muscle dystrophy diseases is correlated with the amount of intramuscular connective tissue in the skeletal muscle. In contrast, myostatin-siRNA-treated masseter muscles show lean tissue with a reduction of intramuscular connective tissue. Furthermore, we also observed a significant downregulation of *CEBPα* and *PPARγ* mRNA expression after Mst-siRNA application. *CEBPα* and *PPARγ* are critical transcription factors in adipogenesis [29], [30]. Taken together, our result indicates that in the masseter muscle of the adipocytes with no inherent myogenic potential may be induced to transdifferentiate into mature myoblasts by Mst-siRNA administration, resulting in the replacement of intramuscular connective tissue with muscle mass.

Skeletal muscles contain various fiber types with different contraction velocities and fatigability characteristics [31]. These fiber types can be classified as type I (slow-twitch) and type II (fast-twitch) fibers on the basis of their predominant myosin heavy chain isoform content [32]. The percentage of type I fibers has been associated with the duty time of the muscle. For the jaw system, it has been found that vertically directed muscles have a larger amount of slow type fibers, which is probably related to their effective action line for jaw closure, generation of occlusal forces and continuation of a mandibular posture [32]. The amount of force produced by skeletal muscles depends on the cross-sectional area when the muscle length is constant [33]. Previously, we investigated the fiber type composition and cross-sectional area of the masseter and digastric muscles of rats and reported a clear heterogeneity in their fiber characteristics [34], consistent with their daily muscle activities recorded by a telemetric EMG recording system [35]. In the present study, to investigate changes in masseter muscle activity after local administration of Mst-siRNA, we measured mouse masseter EMG by a telemetry system. We found that the amount of masseter muscle activity (duty time) in mCAV-3Tg mice was increased by local administration of Mst-siRNA/ATCOL complex irrespective of muscle activity levels. This implies that masseter muscle strength in mCAV-3Tg mice was enhanced through the enlargement of myofiber sizes, indicating a change in muscle fiber type composition. To evaluate the fiber-type transition in the masseter muscle induced by Mst-siRNA application, more studies should be conducted in the future.

It was concluded that atelocollagen-mediated local administration of myostatin-siRNA could lead to masseter muscle hypertrophy and subsequent enhancement of muscular strength in a muscle atrophy disease mouse model (mCAV-3Tg). These results suggest that atelocollagen-mediated application of siRNA is a potential therapeutic tool for future use in treating diseases including muscular atrophy.

## Materials and Methods

### Experimental animals

Sixteen, 24 to 28-week-old mutant caveolin-3 transgenic (mCAV-3Tg) mice, an animal model for muscular dystrophy, were used for the experiments. Mice were kept at a constant ambient temperature (22–24°C) under a constant day-night rhythm and fed on a solid diet *ad libitum*. The experimental protocol described below was approved by the Ethical Committee of the University of Tokushima (Permit number: 21-55).

### Local administration of the Mst-siRNA/ATCOL complex to skeletal muscles of LGMD1C mice

Synthetic 21-nt RNAs were purchased from Koken Co., Ltd. (Tokyo, Japan). The sequences of the siRNAs used are as follows: mouse *GDF-8* (*myostatin*) - 5′-AAGAUGACGAUUAUCACGCUA-3′ and 3′-UUCUACUGCUAAUAGUGCGAU-5′; scrambled control - 5′-AUCGAAUAACCGUAACGUUGA-3′ and 3′-UAGCUUAUUGGCAUUGCAACU-5′. The siRNAs and the ATCOL complexes were prepared as follows. Equal volumes of ATCOL and siRNA solution (siRNA and 1×siRNA buffer, 10 µM final concentration) were combined and mixed by pipetting.

For local administration, the Mst-siRNA/ATCOL complex (100 µl) was introduced into masseter muscles of mCAV-3Tg mice. As a control, the scrambled (scr)-siRNA/ATCOL complex was introduced into contralateral masseter muscles.

### Histometric analysis

The masseter muscle tissues were dissected 2 weeks after Mst-siRNA/ATCOL complex administration. These tissues were frozen in liquid nitrogen-cooled isopentane and sectioned transversely (6 µm) using a cryostat (Leica Microsystems). Frozen sections were stained with hematoxylin eosin and fiber sizes were determined by measuring the area of each transversal myofiber per a fixed area. Approximately 100 myofibers were measured for each tissue sample (about 6–8 fields for each tissue section).

### Real- time quantitative RT-PCR analysis

Total RNA was extracted from the masseter muscle, and reverse transcribed. Transcript levels of *myostatin* were measured using Applied Biosystems 7500 Real time PCR System with SYBR Premix Ex Taq. The specific primers used were as follows: mouse *myostatin*, 5′-CAGCCTGAATCCAACTTAGG-3′ and 3′-CTGAAACCCGAACTGACGCT-5′; *myoD*, 5′-GCGTGCAAGCGCAAGACCAC-3′ and 3′-GATGCGTGGACCTGGCGACG-5′; *myogenin*, 5′-CATGGTGCCCAGTGAATGCAACTC-3′ and 3′-ACCTGTCGTAGTGCCACCTCCTAT-5′; *PPARγ*, 5′-TCAGGCTTCCACTATGGAGTTCA-3′ and 3′-ACTTCCTACGTTCC CAAAAAAG-5′; *CEBPα*, 5′-CGGGCAAAGCCAAGAA GTC-3′ and 3′-TCGTTGCTCATGGCCCATGC-5′; *β-actin*, 5′-CCCTCACGCCATCCTGCGTC-3′ and 3′-CGTCCTCTACCGGTGACGGC-5′

### Telemetric system

Masseter muscle activity was recorded in freely moving animals by a telemetric recording system as applied in previous studies [36]–[39]. Briefly, bipolar electrodes (diameter: 0.45 mm) were connected to implantable 2-channel transmitters for biopotential recording (TL11M2-F20-EET, Data Sciences International [DSI], St. Paul, MN, USA). The distance between the 2 tips of the bipolar electrodes was 1 mm, and the effective electrode tip length was 5 mm. For the implantation of this device, each animal (n = 4, 24∼28-week-old) was anesthetized with intra-abdominal injections of sodium pentobarbital at a dose of 50 µg/g body weight. The transmitter was implanted in the shoulder area, and the two pairs of bipolar electrodes were subcutaneously led to an incision in the right submandibular region. From there, the bipolar electrodes were inserted into the center of the bilateral superficial masseter muscles, and sutured to the muscle surface to prevent them from dislodging. These procedures were done under sterile conditions. From 1 week after recovery of the surgery onwards, muscle activities were continuously recorded over 4 days. The first recording served as a control. Thereafter, the Mst-siRNA/ATCOL complex was locally introduced into masseter muscles. Six and 13 days after local administration of Mst-siRNA/ATCOL complex, muscle activities were recorded for 4 consecutive days, respectively. After the recording period, the animals were sacrificed with an overdose of sodium pentobarbital and the electrode locations were verified by dissection.

In the device, the biopotentials were filtered (1st-order low-pass filter, 158 Hz) and sampled (250 Hz) on the input of each channel. The transmitted data were then collected by a receiver (RPC-1, DSI) placed under the cage. The signals were stored onto a personal computer hard disk, using the Dataquest A.R.T. data acquisition system (DSI).

### Muscle activity analysis

The method of analysis was similar to that performed previously [35], [39]. Briefly, muscle activities recorded for a 24-hour period were visualized using Spike2 software (Cambridge Electronic Design (CED), Cambridge, UK) and analyzed. After motion artifacts had been removed (5 Hz high-pass filter), the signal was rectified and averaged (20 ms window, i.e. 5 samples). To eliminate possible artifacts the 0.001% of the samples (about 43 samples) with the largest amplitudes was excluded. The peak in each of the EMG recordings was defined as the largest of the 99.999% remaining samples indicating the maximum activity for that day and was used for normalization. Activity levels were expressed as percentages of this peak EMG activity [35], [39]. Daily muscle use was characterized by means of the total duration of muscle activity (duty time), which were determined for muscle activities exceeding 5, 20, and 50% of the day's peak activity. A burst was defined as a series of consecutive samples exceeding the aforementioned activity levels [35], [39]. Note that the duty time for activations exceeding a certain level includes the duty times for activations exceeding all higher levels. Duty time exceeding the 5% level was assumed to represent the overall muscle use including all levels and types of muscle activities. Muscle activity exceeding 50% of the peak EMG level was considered as a representative for the most forceful muscle usage.

### Statistical analysis

All data are expressed as means ± standard deviation (SD). The daily duty time was averaged for each activity level. Analysis of variance (ANOVA) was used to monitor for significant differences in daily duty time exceeding 5%, 20% and 50% levels. If the ANOVA was significant, the Boneferroni/Dunn procedure was used as a post-hoc test. Data from real-time PCR and morphometric analyses were subjected to an unpaired Student's t-test. A P-value of 0.05 or less was considered statistically significant.
